# T1rho imaging of head and neck cancer: its association with pathological and immunohistochemical biomarkers in nasopharyngeal carcinoma

**DOI:** 10.1186/s40644-025-00940-7

**Published:** 2025-10-21

**Authors:** Qi Yong H. Ai, Amy BW Chan, Angela Z. Chan, Joyce Kwok Wing Lam, Ho-Sang Leung, Ziqiang Yu, Frankie KF Mo, Lun M. Wong, Weitian Chen, Ann D. King

**Affiliations:** 1 Department of Diagnostic Radiology, The University of Hong Kong, LG3-08, Jocky Club Building for Interdisciplinary Research, 5 Sassoon Road, Pokfulam, Hong Kong S.A.R., China; 2 Department of Imaging and Interventional Radiology, The Chinese University of Hong Kong, Prince of Wales Hospital, Shatin, New Territories, Hong Kong S.A.R., China; 3 Department of Anatomical and Cellular Pathology, The Chinese University of Hong Kong, Prince of Wales Hospital, Shain, New Territories, Hong Kong S.A.R., China; 4 Department of Clinical Pathology, North District Hospital, Hospital Authority, Tuen Mun, New Territories, Hong Kong S.A.R., China; 5 Histopathology and Cytology Division, Public Health Laboratory Services Branch, Health Protection, Department of Health, Hong Kong Government, Kowloon, Hong Kong S.A.R., China; 6 Department of Clinical Oncology, Li Ka Shing Institute of Health Sciences, State Key Laboratory of Translational Oncology, The Chinese University of Hong Kong, Prince of Wales Hospital, Shatin, New Territories, Hong Kong S.A.R., China

**Keywords:** T1rho imaging, Diffusion weighted imaging, Nasopharyngeal carcinoma, Histopathological biomarkers, Immunohistochemical

## Abstract

**Purpose:**

T1rho imaging showed potential applications in cancer imaging but little research explored the underlying biological processes that contribute to the T1rho values in cancer. This study aimed to investigate the potential associations between quantitative imaging biomarkers from T1rho imaging and the well-established diffusion weighted imaging (DWI), with tumour-stromal, immunohistochemical (IHC), and tumour-infiltration-lymphocytes (TIL) biomarkers in nasopharyngeal carcinoma (NPC).

**Methods:**

Pre-treatment T1rho and DWI imaging of primary NPCs were performed in 50 prospectively recruited patients. The mean T1rho and apparent diffusion coefficient (ADC) of NPC were obtained and correlated with tumour-stromal, IHC, TIL biomarkers using the Pearson Correlation test and the coefficients (R) were calculated.

**Results:**

The mean T1rho values negatively correlated with collagenous stroma-lymphoid stroma (*R*=-0.314, *p* = 0.03) and positively correlated with percentage of tumour cells positive for Ki-67 (*R* = 0.402, *p* < 0.01), but there were no associations between T1rho values and the other tumour-stromal, IHC or TIL biomarkers (*p* = 0.16–0.98) or between ADC values and any of these biomarkers (*p* = 0.07–0.82).

**Conclusion:**

Our results showed the possible underlying biological mechanisms of T1rho imaging in head and neck cancer. T1rho imaging negatively correlated with the ratio of collagenous to lymphoid stroma, and positively correlated with tumour cell proliferation, which are both known to be predictors of outcome, suggesting that T1rho imaging may have a valuable role in head and neck cancer imaging. As this is a preliminary study with small sample size, further studies are encouraged to validate our findings.

## Introduction

A range of quantitative MRI techniques, including diffusion weighted imaging (DWI), dynamic contrast enhanced MRI and chemical exchange saturation shift transfer imaging, have been evaluated for the non-invasive evaluation of the tumour microenvironment and have been used successfully to predict and monitor the response of cancers to radiotherapy and chemotherapy. T1rho imaging, which has been applied to clinical research for two decades, is a new quantitative MRI sequence for cancer imaging. T1rho imaging assesses spin-lattice relaxation in a rotation frame and measures the decay of magnetization in the transverse plane during a spin-lock pulse. This technique does not request intravenous injection of contrast enhancement agents and can be easily adapt to the routine MRI protocol. Recently, our team overcomes the sensitivity to the B_1_ RF and B0 field inhomogeneity which greatly improved image quality compared to other non-contrast enhanced quantitative MRI sequences [1, 2]. T1rho imaging is sensitive to fast exchanging protons and chemical exchange in biological tissues and is also sensitive to protein denaturation. T1rho values have been linked to high levels of glycosaminoglycans/proteoglycans which are also known to regulate many cancer pathways, including collagen fibrillogenesis [3–5]. T1rho values therefore may potentially provide new imaging biomarkers to assess these macromolecules in the cancer extracellular matrix (ECM). Early cancer studies suggest T1rho imaging may have a role in cancer treatment monitoring [6, 7], including in the head and neck [7] where it also has been shown to have good test-retest repeatability of T1rho [8] and ability to distinguish benign from malignant tissues used to characterise tissues [9]. However, no studies have directly explored the associations T1rho values and underlying biological processes in head and neck cancer.

In this study we correlated the T1rho values of primary nasopharyngeal cancers (NPC) with biomarkers that have been shown to influence treatment response and prognosis. We correlated the mean T1rho values with their tumour-stromal content based on ratio of tumour-stroma (TSR), tumour-collagenous stroma (TCSR) and collagenous stroma-lymphoid stroma (CSLSR). We also correlated the mean T1rho values with immunohistochemical (IHC) biomarkers in which overexpression is related to treatment resistance and poor outcome in head and neck cancers. These included programmed death ligand-1 (PD-1), where overexpression also increases sensitivity to the PD-L1-related immunotherapy [10–12], hypoxia inducible factor- 1-alpha (HIF-1a) which reflects tumour hypoxia [13] and Ki-67 which reflects the cell proliferation activity and is also associated with an increased risk of nodal metastases [14–16]. In addition, we evaluated T1rho values with tumour-infiltration lymphocytes (TILs) which are associated with a more favourable outcome [17].

## Materials and methods

### Participants

**Fig. 1:**
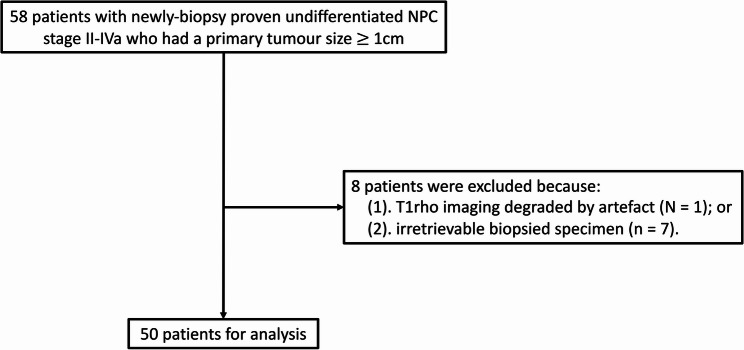
shows a flowchart for patient selection

This prospective study was performed as part of a T1rho study to predict treatment response [7] with local institutional board approval (The Joint of The Chinese University of Hong Kong and New Territories East Cluster Clinical Research Ethics Committee. Reference number: CRE-2019.026). Written informed consent was obtained from each eligible patient. Patient recruitment for the study followed the inclusion criteria: (1) adult patients with newly biopsy-proven undifferentiated NPC; (2) pre-treatment staging MRI showed stage II – IVa NPC and (3) size of primary NPC ≥ 1 cm in the axial plane. The first 50 patients with retrievable biopsy specimens and T1rho images not degraded by artefact were included in the study. A flowchart for patient selection is shown in Fig. 1. Forty-one out of 50 patients were previously reported in a study which investigated the role of T1rho imaging in the prediction of treatment response in NPC [7], and this study investigated the potential biological processes that contribute to T1rho imaging in cancer. The study was conducted in full accordance with the Declaration of Helsinki 2013 and its later amendments.

### MRI acquisition

#### T1rho imaging and DWI

MRI was performed on a Philips Achieva TX 3 T scanner (Philips Healthcare, Best, The Netherlands) with a body coil for radiofrequency transmission and a 16-channel Philips neurovascular phased-array coil for reception. Patients underwent pre-treatment MRI with T1rho, DWI and anatomical imaging.

T1rho imaging was performed using adiabatic continuous wave constant amplitude spin-lock approach [2] followed with single shot turbo spin echo acquisition. The spin-lock radiofrequency pulse cluster consisted of a constant amplitude spin-lock radiofrequency pulse sandwiched by an adiabatic half passage (AHP) and a reverse adiabatic half passage (rAHP). Hyperbolic secant pulses were used as the AHP and rAHP with the B1 amplitude of the AHP and rAHP set equal to that of the spin-lock radiofrequency pulse [1, 2]. In summary the imaging parameters were: repetition time/echo time 2500/15 msec; field of view, 230 mm × 216 mm; resolution, 1.2 mm × 1.2 mm; slice thickness, 4 mm; number of slices, 5–9; sensitivity encoding factor, 2; AHP and rAHP duration, 25 msec; the maximum amplitude of frequency waveform modulation of the AHP and rAHP: 400 Hz; coefficient factor β for AHP and rAHP, 4; frequency of spin-lock, 400 Hz; and spin lock time, 0, 10, 30, 55, and 90 msec (full details of the pulse sequence are reported previously [18]). The total T1rho imaging scan time was 1 min and 10 s to 1 min and 50 s.

DWI was acquired using a fat-suppressed, single-shot spin-echo echo-planar imaging sequence. The imaging parameters were: repetition time/echo time, 2000/50 mesc; field of view, 230 mm × 230 mm; resolution, 1.7 mm × 2.1 mm; slice thickness, 4 mm; number of slices: 9; echo train length: 55; sensitivity encoding factor: 2; number of signals acquired: 4; and 6 b-values (0, 200, 400, 600, 800 and 1000 s/mm^2^). The total DWI scan time was 1 min 30 s.

Anatomical MRI sequences included at least (1) axial T1-weighted image (T1WI), (2) axial fat-suppressed T2-weighted image (FST2WI), (3) coronal T1WI and (4) contrast-enhanced T1WI with and without fat suppression in the pre-treatment scan.

### Imaging analysis

T1rho images were reconstructed at matrix size 288 × 288. These images were used for T1rho quantification using an in-house Matlab (Mathworks) program. The images were smoothed by a sliding 2 × 2 window throughout the image before quantification. At each pixel, the image intensity was fitted to the relaxation model [8, 9] to calculate the T1rho value. We used a variant of the dichotomy method [19] to fit the data to this relaxation model to quantify T1rho values. This condition was incorporated in the fitting algorithm to improve fitting accuracy. The peak signal-to-noise ratio (PSNR) was calculated to evaluate the goodness of fit [8, 9]. Criteria were set to exclude pixels with obvious errors or possible unreliable fitting results. A pixel was excluded from final analysis if it had PSNR < 30 or an extreme T1rho value (< 15 msec or >200 msec).

Olea Sphere (version 3.0; Olea Medical SA) was used for the diffusion post-processing steps by implementing a Bayesian probability-based algorithm using 6 b-values (0, 200, 400, 600, 800, 1000 s/mm^2^) to fit a mono-exponential diffusion model to calculate the conventional apparent diffusion coefficient (ADC).

To accurately correlate the T1rho and ADC values with the pathological and IHC biomarkers of the biopsy sample, a round-shape ROI with a size of 0.5 cm in diameter was manually placed on the superficial primary NPC on the same side as the biopsy, positioned one slice above or below the biopsy site on the T1rho and ADC maps. Any necrotic or cystic areas with reference to the corresponding anatomical images were excluded from the ROIs. The ROIs were performed by a researcher with 8 years of experience in MRI of NPC (Q.Y.H.A., observer 1) and by an on-board radiologist with 3 years of experience in head and neck radiology (H.S.L. observer 2). The mean values obtained from the two observers were averaged for the analysis. The mean values of T1rho and ADC were calculated for further analysis.

### Pathological and IHC staining and analysis

#### Pathological and IHC staining

Tissues were isolated and fixed for 24 h in 10% neutral-buffered formalin for subsequent tissue processing and embedded in paraffin (FFPE). The FFPE sections of 4 μm thickness were prepared and mounted on uncoated glass slides for haematoxylin and eosin (H&E) and coated slides for immunohistochemistry staining. Briefly, the tissues were stained using an automated slide stainer (Benchmark Ultra; Ventana Medical Systems, Roche, Switzerland) for CD3 (1:80) (#NCL-L-CD3-565, Clone: LN10, Novocastra, United Kingdom), CD4 (1:3) (#790–4423, Clone: SP35, Ventana, United Kingdom) CD8 (1:300) (#M7103, Clone: C8/144B(1), Dako, United States), PD-L1 (#SK006, Clone: 22C3, Dako, United States), HIF-1α (1:100) (#610959, Clone: 54/HIF-1α, BD Biosciences, United States) and Ki67 (1:200) (#RM-9106-S, Clone: SP6, Thermo Scientific, United States). Heat-induced epitope retrieval was performed using EDTA buffer for markers CD3, CD4 and, CD8. For PD-L1, epitope retrieval was performed using PT Link EnV FLEX TRS High pH, United States, as per manufacture’s protocol. For HIF-1α and Ki67, epitope retrieval was performed using the Ventana Cell Conditioning Solution 1, United States, as per manufacturer’s protocol. Chromogenic immunodetection of our targets were performed using Optiview DAB IHC Detection kit, Roche, United States (CD3), #DS9800 Bond Polymer Refine Detection Kit, United States (CD4 and CD8), Dako PD-L1 IHC 22C3 pharmDX Kit, United States (PD-L1), and OptiView DAB IHC Detection Kit plus Amplification Kit, United States (Ki67), as per manufacture’s protocol.

#### Analysis of the tumour-stromal, IHC and TIL biomarkers

Two pathologists (C.B.W. and A.Z.C.) with 20 years and 5 years of experience in head and neck pathology, respectively, independently reviewed the cases. They were blinded to the clinical details to ensure unbiased interpretation of the tumour morphology and immunohistochemical staining results. The tumour-stromal biomarkers which included ratios of (1) the total tumour area to the total stromal area (tumour-stroma-ratio, TSR), (2) the total tumour area to the total area of fibrotic stroma (tumour-collagenous stroma-ratio (TCSR), and (3) total area of fibrotic stroma to the total area of lymphoid stroma (collagenous stroma-lymphoid stroma-ratio, CSLSR) were assessed and expressed as numerical values to two decimal places. The IHC biomarkers, which included percentages of tumour cells positive for PD-L1 (PD-L1%), HIF-1α (HIF-1α%), and Ki-67 (Ki-67%) was semi-quantified and expressed as a percentage of the total tumour cell population, with the values rounded to the nearest 5%. Background lymphoid cells were excluded in the assessment. Additionally, the percentages of CD3+, CD4+, and, CD8 + TILs were separately evaluated for the intratumoural compartment and stromal compartments (CD3 + intra%, CD4 + intra% CD8 + intra%, CD3 + stroma%, CD4 + stroma% CD8 + stroma%), with these values also rounded to the nearest 5%. All scoring was conducted using an Eclipse Ci microscope (Nikon, Japan).

### Tumour staging

The primary tumour and nodal metastases on the pre-treatment MRI were staged based on the 9th version of American Joint Committee on Cancer (AJCC) Cancer Staging Manual [20].

### Statistical analysis

Imaging markers (mean T1rho and mean ADC values) were correlated with tumour-stromal biomarkers (TSR, TCSR and CSLSR), IHC biomarkers of the tumour component (PD-L1%, HIF-1α%, Ki-67%), and the percentages of TILs in the intratumoural and tumour-stromal components (CD3 + intra%, CD4 + intra% CD8 + intra%, CD3 + stroma%, CD4 + stroma% CD8 + stroma%) using the Pearson correlation test, and the Pearson correlation coefficient (R) was calculated. Intra-observer agreement for T1rho and ADC values were evaluated using the intra-class correlation test and the intra-class coefficient (ICC) was calculated. All of the statistical tests were 2-sided, and a *p*-value < 0.05 was considered to indicate a statistically significant difference. Analyses were performed using the statistical analysis software SPSS (version 26.0; IBM, NY, USA).

## Results

### Participants

There were 58 eligible patients prospectively recruited for the study. After excluding 8 patients: (1) degraded by artefact (*n* = 1) or (2) biopsy specimen was irretrievable (*n* = 7), there were 50 specimens from 50 patients for the analysis (Fig. 1) in patients scanned between June 2020 and September 2022. Patient demographics and cancer staging are shown in the Table 1.

**Table 1:** Patient demographics, and cancer staging

	Patients (%) *N* = 50
Age (year)	
Median Range	56 25–77
Sex	
Male Female	34 (68.0%) 16 (32.0%)
T stage	
T1 T2 T3 T4	11 (22.0%) 8 (16.0%) 27 (54.0%) 4 (8%)
N stage	
N0 N1 N2 N3	7 (14.0%) 18 (36.0%) 12 (24.0%) 13 (26.0%)
Primary NPC short axis diameter (mm)	
Median	18.0
Range	10.6–39.4

*NPC* nasopharyngeal carcinoma

### Correlation of mean T1rho and ADC values with tumour-stromal biomarkers, IHC biomarkers and TILs in NPC

Table 2 shows the median, interquartile values and ranges of the imaging markers, tumour-stromal, IHC and TIL biomarkers of the primary NPC. Tumour-stromal contents were identified on all specimens, of which 30/50 (60.0%) only identified lymphoid stromal content, and 20/50 (40.0%) identified both lymphoid and collagenous stromal contents. All specimens were positive for PD-L1, HIF-1α and Ki-67 biomarkers and all TIL biomarkers, of which 9/50 (18.0%), 10/50 (20.0%), 1/50 (2.0%), and 2/50 (4.0%) NPC specimens showed less than 5% of cells positive for the PD-L1, HIF-1α, CD3 + intra, and CD8 + stroma, respectively.

**Table 2:** Imaging and histopathological biomarkers in primary NPC

Imaging biomarkers	Median (Interquartile)	Range
T1rho value *(msec)*	67.6 (63.4, 72.0)	53.7–81.7
ADC value *(x* 10^−3^ mm^2^/s*)*	0.80 (0.72, 0.87)	0.63–1.13
Tumour-stromal biomarkers		
TSR	1.5 (0.7, 10.0)	0.05–60.0
TCSR	2.5 (0, 20.0)	0–100.0
CSLSR	0.1 (0, 0.3)	0–0.3
IHC biomarkers		
PD-L1%	10 (5, 40)	< 5–90
HIF-1α%	10 (5, 20)	< 5–60
Ki-67%	50 (30, 70)	10–90
TIL biomarkers		
CD3 + intra%	20 (15, 40)	< 5–60
CD4 + intra%	20 (10, 30)	5–60
CD8 + intra%	15 (10, 25)	5–40
CD3 + stroma%	80 (70, 90)	10–90
CD4 + stroma%	50 (30, 60)	5–90
CD8 + stroma%	30 (15, 40)	< 5–70

*ADC* apparent diffusion coefficient, *CSLSR* collagenous stroma-lymphoid stroma-ratio, *HIF-1a* Hypoxia inducible factor- 1-alpha, *IHC* immunohistochemistry, *NPC* nasopharyngeal carcinoma, *PD-L1* programmed death ligand-1, *TCSR* tumour-collagenous stroma-ratio, *TIL* tumour-infiltration lymphocyte, *TSR* tumour-stroma-ratio

**Fig. 2:**
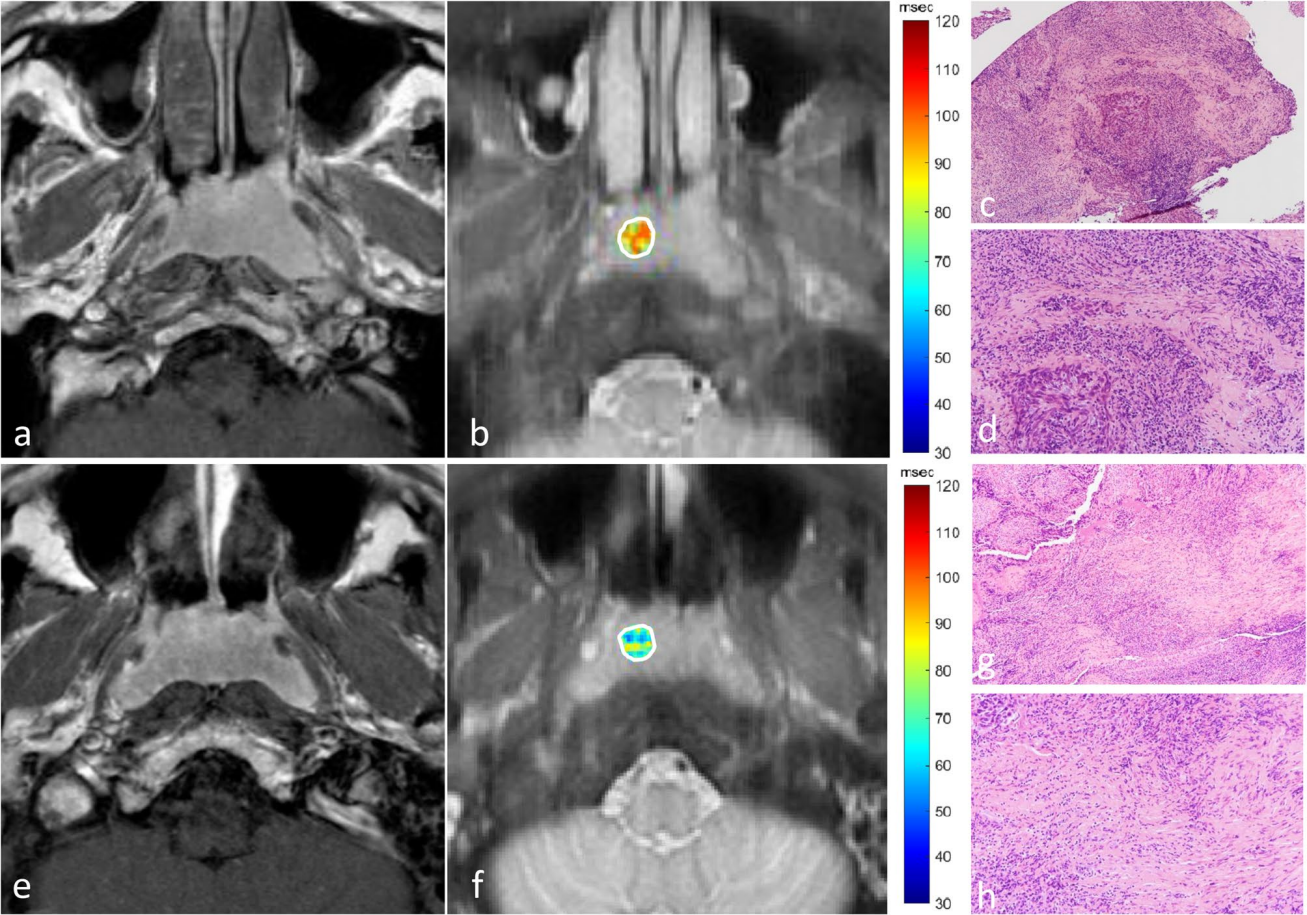
shows axial contrast-enhanced T1-weighted MR images (**a** and **e**), T1rho maps (**b** and **f**) and H&E-stained specimens on 10-fold magnification (**c** and **g**) and on 20-fold magnification (**d** and **h**) of representative patients who had NPC with low collagenous stroma-lymphoid stroma ratio (CSLSR) (**a**-**d**) and with high CSLSR (**e**-**h**). The patient with a low CSLSR of 0.05 on the H&E-stained specimen (**c** and **d**) showed a T1rho mean value of 77.3 msec extracted from the primary NPC (white contour) on the T1rho map (**b**); and the patient with a high CSLSR of 1.20 on the H&E-stained specimen (**g** and **h**) showed a T1rho mean value of 66.4 msec extracted from the primary NPC (white contour) on the T1rho map (**f**). Overall, the T1rho mean value negatively correlated with CSLSR in NPC specimens (Pearson correlation coefficient = −0.314, *p* < 0.03). Abbreviations: *CSLSR* collagenous stroma-lymphoid stroma-ratio, *NPC* nasopharyngeal carcinoma

**Fig. 3:**
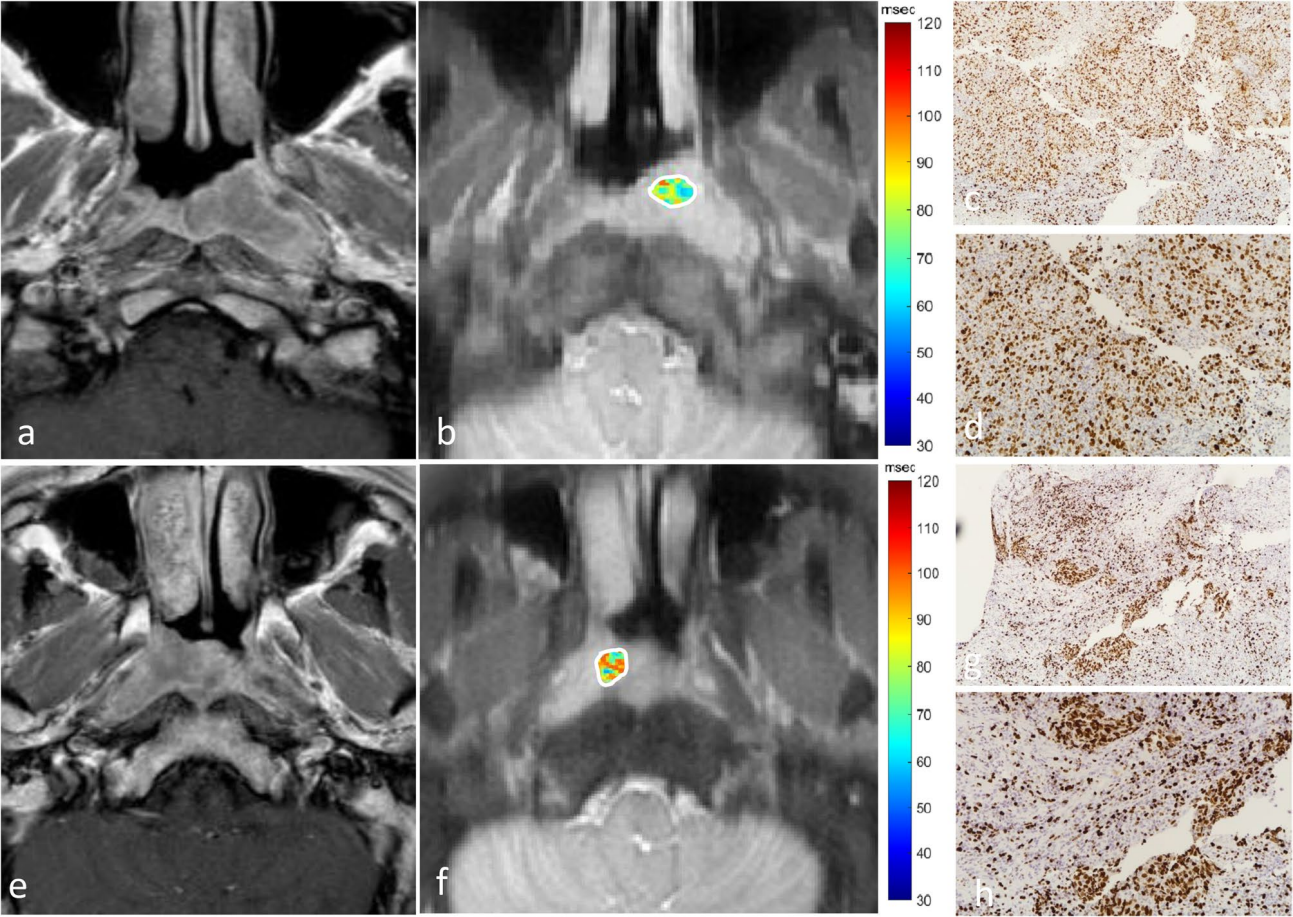
shows axial contrast-enhanced T1-weighted MR images (**a** and **e**), T1rho maps (**b** and **f**) and Ki-67-stained specimens on 10-fold magnification (**c** and **g**) and on 20-fold magnification (**d** and **h**) of representative patients who had NPCs with high (**a**-**d**) and low (**e**-**h**) percentages of tumour cells positive for Ki-67. The patient with a low Ki-67% of 30% on the Ki-67-stained specimen (c and d) showed a T1rho mean value of 67.5 msec extracted from the primary NPC (white contour) on the T1rho map (**b**); and the patient with a high Ki-67% of 80% on the Ki-67-stained specimens (**g** and **h**) showed a T1rho mean value of 73.8 msec extracted from the primary NPC (white contour) on the T1rho map (**f**). Overall, the T1rho mean value positively correlated with Ki-67% in NPC specimens (Pearson correlation coefficient = 0.402, *p* < 0.01). Abbreviations: *NPC* nasopharyngeal carcinoma

Regarding the tumour-stromal biomarkers, T1rho value negatively correlated with CSLSR (*R* = −0.314, *p* = 0.03) (Fig. 2), but not TSR (*p* = 0.50) and TCSR (*R* = 0.38) (Table 3); regarding the IHC biomarkers, T1rho value positively correlated with Ki-67% (*R* = 0.402, *p* < 0.01) (Fig. 3), but did not correlate with PD-L1% (*p* = 0.98) and HIF-1α% (*p* = 0.25) (Table 3); T1rho value did not correlate with any of the TIL biomarkers (*p* = 0.16 to 0.83) (Table 3). The ADC value did not correlate with any tumour-stromal, IHC and TIL biomarkers (*p* = 0.07 to *p* = 0.82) (Table 3).

**Table 3:** Correlation of imaging markers with IHC biomarkers in primary NPC in 50 patients

	Imaging markers			
	T1rho value		ADC value	
	R	P-value	R	P-value
Tumour-stromal biomarkers				
TSR	−0.099	0.50	0.070	0.63
TCSR	0.127	0.38	−0.200	0.16
CSLSR	−0.314	**0.03**	−0.102	0.48
IHC biomarkers				
PD-L1%	0.004	0.98	−0.201	0.16
HIF-1α%	0.165	0.25	0.189	0.19
Ki-67%	0.402	**< 0.01**	0.033	0.82
TIL biomarkers				
CD3 + intra%	−0.075	0.60	0.119	0.41
CD4 + intra%	−0.202	0.16	0.257	0.07
CD8 + intra%	−0.132	0.36	−0.055	0.70
CD3 + stroma%	0.092	0.53	−0.049	0.74
CD4 + stroma%	−0.031	0.83	0.160	0.27
CD8 + stroma%	−0.080	0.58	0.165	0.25

*ADC* apparent diffusion coefficient, *CSLSR* collagenous stroma-lymphoid stroma-ratio, *HIF-1a* Hypoxia inducible factor- 1-alpha, *IHC* immunohistochemistry, *NPC* nasopharyngeal carcinoma, *PD-L1* programmed death ligand-1, *TCSR* tumour-collagenous stroma-ratio, *TIL* tumour-infiltration lymphocyte, *TSR* tumour-stroma-ratio

The analysis of inter-observer agreement showed an ICC of 0.83 for T1rho value and 0.84 for ADC value.

## Discussion

T1rho imaging is a new functional MRI sequence for cancer imaging and this is one of the first studies to investigate the associations between quantitative T1rho measurements and the biological processes in the cancer microenvironment. In this head and neck cancer study we correlated mean T1rho values with the stroma in primary NPCs. Our results showed that lower T1rho values were associated with a higher CSLSR; that is a higher percentage of the collagen compared to lymphoid cells in the cancer stroma. However, it is uncertain if this finding directly reflects the collagen content. Magnetization transfer (MT) effect is associated with collagen, but this effect is not significant in the on-resonance techniques used for T1rho imaging, and while our T1rho sequence has some off-resonance spin-lock effects, which can elevate sensitivity to MT effect, these are also unlikely to be significant. Moreover, in non-cancerous T1rho studies, both negative [21, 22] and positive associations [23, 24] have been reported with collagen content. This discrepancy may be due to the sensitivity of T1rho to multiple physical effects including water content change, which can confound its characterization of macromolecule content change [25]. The underlying biological processes for our negative correlation of T1rho values with collagenous content is therefore not completely understood. The CSLSR was the only biomarker ratio that focused solely on the tumour-stromal content without any influence from tumour cells, which is a possible explanation for why this was the only biomarker ratio that correlated with T1rho.

Our study also correlated T1rho values with IHC and TIL cancer biomarkers, that are known to have an important influence on treatment response and prognosis. The mean T1rho values correlated positively with the tumour proliferation marker Ki-67%. As T1rho is known to reflect mainly macromolecules in the ECM, one possible explanation for this observation is that high tumour proliferation activates complex pathways in the ECM related to the collagenous content and production of proteoglycans [26–28]. None of the other markers that reflected PD-L, tumour hypoxia or tumour-infiltrating lymphocytes were associated with our T1rho values.

It is worthy to note that this study was designed as a preliminary study to understand the potential underlying biological processes of T1rho imaging in cancer imaging. Therefore, the current findings are yet ready for clinical practice. However, the findings are inspiring, suggesting that T1rho imaging has the potential to serve as an imaging biomarker for identifying patients high Ki-67 NPC, who may benefit from targeted immunotherapy in future treatment. Furthermore, since CSLSR and Ki-67% are predictors of long-term outcome in NPCs [14–16], our results indicated that T1rho imaging may have the potential to help to stratify patients based on their risk of disease recurrence without additional pathological and IHC examinations.

We also explored the associations of pathological and IHC features with ADC values obtained from DWI, which is the most established functional MRI sequence in the head and neck cancer. The biological processes that contribute to DWI in cancer imaging have been more widely investigated. Driessen JP et al. showed that ADC mean values positively correlated with tumour-stromal content in the head and neck cancer [29], which would explain why head and neck squamous cell carcinoma with high ADC mean value had poorer outcome than those with lower ADC mean values [30–32], but for other biomarkers there are conflicting results. Negative correlations are reported between ADC mean values and PD-L1% [33, 34], and Ki-67% [34–37] while other studies failed to show any correlation [38, 39]. No associations have been reported between ADC mean values and HIF-1α% or TIL biomarkers [35, 40, 41], but ADC entropy has been linked to TIL [41]. In this study we also found no corelations between ADC and any of the IHC or TIL markers or tumour-stromal ratios.

This study had limitations. First, since quantitative analysis of the percentage of the cells for these histopathological biomarkers is time-consuming and laborious, similar to other head and neck studies [42–45] we used semiquantitative analysis for the histopathological biomarker analysis. We believe, the assistance of artificial intelligence [46], the quantitative analysis of the histopathological biomarkers will be more easily performed in the future. Second, this study was limited to correlation with only the most relevant histopathological biomarkers in this cancer. Third, we did not include non-NPC patients in the analysis and so it is unknown whether the correlation of T1rho values with CSLSR is only applied to patients with cancer or more general to those who without NPC as well. In addition, since patients with NPC are not routinely treated with surgical dissection, pathological and IHC features of the entire primary tumour were unavailable for analysis. To reduce large discrepancies in tumour heterogeneity between the biopsied NPC and T1rho imaging, we extracted T1rho and ADC values only from a 0.5 cm round-shape ROI on the primary tumour, on the same side as the biopsy, and positioned one slice above or below the biopsy site. Nevertheless, studies correlating T1rho imaging with pathological and IHC features using larger biopsy samples are recommended.

## Conclusion

T1rho imaging is a new quantitative functional MRI technique for cancer imaging, and this study correlated T1rho values with the biological processes in head and neck cancer that are known to influence treatment outcome. Our study of NPC showed that T1rho imaging is sensitive to the macromolecules in the ECM which contribute to the stromal content. Low T1rho values correlated with a high ratio of collagenous stroma to lymphoid stroma, although it is unclear if this is directly related to collagen content, and high T1rho values correlated with high positivity for Ki-67%, a biomarker for tumour cell proliferation. Both these markers influence treatment response and prognosis in NPC, suggesting possible mechanisms by which T1rho imaging could predict and monitor head and neck cancer treatment response. No correlations were found between T1rho and PD-L1, tumour hypoxia or tumour-infiltrating lymphocytes or between DWI, using the mean ADC, and any of the tumour-stromal, IHC, or TIL biomarkers. As this is a preliminary study with small sample size, further studies are encouraged to validate our findings.

## Data Availability

The datasets generated or analysed during the study could be available one reasonable request to the corresponding author.
